# Benchmarking AlphaMissense against ClinVar for Diagnostic Interpretation of Missense Variants in Inherited Retinal Diseases

**DOI:** 10.1016/j.xops.2025.100997

**Published:** 2025-11-10

**Authors:** Mark Lindquist, Samson Darrah, Stefan T. Stafie, Debarshi Mustafi

**Affiliations:** 1 Department of Ophthalmology, University of Washington, Seattle, Washington; 2 Roger and Angie Karalis Johnson Retina Center, Seattle, Washington; 3 Department of Bioengineering, University of Washington, Seattle, Washington; 4 Virginia Merrill Bloedel Hearing Research Center, Seattle, Washington; 5 Brotman Baty Institute for Precision Medicine, Seattle, Washington; 6 Division of Ophthalmology, Seattle Children’s Hospital, Seattle, Washington

**Keywords:** AlphaMissense, ClinVar, Hypomorphic variants, Inherited retinal diseases, Variant prediction

## Abstract

**Purpose:**

AlphaMissense is a newer deep learning–based variant predictor that evaluates the structural consequences of missense variants, the most common pathogenic variant type in inherited retinal diseases (IRDs). This study evaluates the diagnostic utility of AlphaMissense in IRDs by assessing its concordance with ClinVar annotations and exploring how other variant-level metrics may refine its predictions.

**Design:**

A cross-sectional benchmarking study using public variant resources, with a single illustrative clinical case.

**Participants:**

Missense variants from 107 IRD genes; 1 patient case undergoing long-read sequencing.

**Methods:**

Pathogenicity scores from AlphaMissense were extracted from 128 248 variants present in both IRD genes and the Genome Aggregation Database. Among these, 4204 had definitive ClinVar classifications and were used to calculate AlphaMissense specificity, sensitivity, and false discovery rate (FDR). Population-based metrics, including allele frequency, homozygote count, and Combined Annotation Dependent Depletion score, were analyzed to identify salient features that would be associated with discordance. Long-read sequencing was carried out in a monoallelic *ABCA4* patient with late-onset macular dystrophy for phased variant analysis.

**Main Outcome Measures:**

Concordance between AlphaMissense predictions and ClinVar annotations was used to calculate sensitivity, specificity, and FDR. Variant-level metrics between discordant variants. Case-based reclassification of hypomorphic variants with long-read sequencing.

**Results:**

AlphaMissense achieved a specificity of 94.1% and sensitivity of 79.4% in IRD genes, with specificity reaching 100% in A*BCA4, USH2A, RPGR, and PRPH2*, which are 4 of the most common IRD genes. The FDR was 9.6%. AlphaMissense underperformed in predicting hypomorphic variants, particularly in *ABCA4*-associated Stargardt disease. Variant-level metrics were effective in identifying false negatives. In a clinical case, phased variant analysis identified a potential hypomorphic *ABCA4* variant predicted as benign by AlphaMissense.

**Conclusions:**

AlphaMissense demonstrates high specificity for pathogenicity prediction in IRD-associated genes; however, its reduced sensitivity, as seen in hypomorphic variants, suggests a need to incorporate population and functional metrics scores, which may improve classification accuracy, especially as long-read sequencing enables phased variant analysis.

**Financial Disclosures:**

The authors have no proprietary or commercial interest in any materials discussed in this article.

Inherited retinal diseases (IRDs) are a heterogeneous group of genetic disorders characterized by dysfunction of photoreceptor cells and the retinal pigment epithelium. Inherited retinal diseases affect approximately 2 million people worldwide and are a leading cause of blindness in the working-age population.^1^ This leads to progressive vision loss beginning in childhood and resulting in lifelong visual impairment. This can have a significant impact on the patient’s quality of life.^2^ Large-scale genetic testing has provided molecular insight into the molecular causes of IRDs,^3^ yet a substantial proportion of patients with clinical features of IRDs receive nondiagnostic genetic results due to the inability to assign pathogenicity to detected variants in IRD genes.^4^

A major contributor to these inconclusive results is the challenge of interpreting missense variants, which are particularly common in IRD-associated genes. Unlike nonsense or frameshift variation that clearly disrupts protein function, missense variants, which are the most prevalent form of variation in the human genome,^5^, ^6^, ^7^ cannot be confidently interpreted from sequence changes alone. Their functional impact depends not only on the biochemical nature of the amino acid substitution but also on its location within the protein and its evolutionary conservation. Consequently, many missense variants are classified as variants of undetermined significance (VUS), or they are misclassified as pathogenic or benign.^8^ Assigning pathogenicity to VUS currently requires integrating genomic data, clinical phenotype, segregation analysis and functional assays. However, these methods are resource intensive and not feasible for all genes. Functional predictions from tools such as Variant Effect Predictor and Combined Annotation Dependent Depletion (CADD) scores are limited and incomplete functional annotations of reference databases restrict their clinical utility.^8^ Hypomorphic missense variants, which lead to partial loss of protein function, are an additional challenging subset of variants in IRDs to classify.^9^ These variant types often result in milder or variable phenotypes with later onset, making them particularly difficult to interpret based on sequence information alone. This uncertainty leads many hypomorphic variants to be categorized as VUS and ultimately limits the ability of genetic testing to deliver a definitive diagnosis and complicates patient management.

There is a clear need for tools that can more accurately predict pathogenicity of missense variants in IRDs to improve molecular diagnostics and better guide clinical decision-making. The deep learning model, AlphaMissense, has emerged as a promising solution.^10^ It integrates 3-dimensional protein structure predictions from AlphaFold2,^11^^,^^12^ evolutionary conservation, and population variant frequency data to generate a pathogenicity probability score for every possible missense variant across the human proteome.^13^ Whereas previous studies have compared AlphaMissense predictions with ClinVar classifications,^14^^,^^15^ none have specifically evaluated its diagnostic performance in IRD-associated genes. To address this gap, we evaluated the ability of AlphaMissense predictions in the most commonly implicated IRD genes. We also investigated whether incorporating additional variant-level metrics, such as allele frequency, homozygote count, and CADD score, could refine its predictive performance. Our findings demonstrate that AlphaMissense has high specificity but lower sensitivity in predicting pathogenicity in IRD genes based on ClinVar annotations. This suggests that while AlphaMissense reliably identifies pathogenic variants, it may misclassify some benign or uncertain variants. Finally, we show that incorporating particular variant-level metrics (allelic frequency, homozygote count, and CADD score data) can improve detection of potential misclassifications, highlighting a method for more accurate missense variant interpretations in IRDs in the future.

## Methods

### Selection of Genes

A total of 107 IRD genes associated with IRDs were selected based on their frequent inclusion in clinical IRD panels such as MyRetinaTracker, their listing in the Retinal Information Network, and their documentation in peer-reviewed IRD gene reviews. The complete list of genes analyzed in this study is provided in Supplementary Table 1 (available at www.ophthalmologyscience.org).

### AlphaMissense Variant Dataset and Annotation

Missense variant pathogenicity scores were obtained from the precomputed AlphaMissense dataset, which is publicly available through the official repository of DeepMind (https://alphamissense.hegelab.org/). AlphaMissense provides predictions for 216 million single amino acid substitutions, based on Ensembl gene models from the GRCh38 human genome assembly.^10^ Each entry includes the gene symbol, Ensembl protein ID, amino acid position, reference and alternate residues, and a pathogenicity score ranging from 0 to 1. AlphaMissense scores were computed for a single representative transcript for each gene. Pathogenicity scores were interpreted using established thresholds: variant scores from 0.000 to 0.333 were classified as “likely benign,” 0.334 to 0.564 as “ambiguous,” and 0.565 to 1.000 as “likely pathogenic.”^10^ Variants in the ambiguous range were not included in the primary sensitivity and specificity calculations because this interval represents low-confidence predictions that fall outside the high-precision thresholds defined by the model developers. Including these predictions in binary classification would require arbitrary assignment as benign or pathogenic and could bias performance estimates. To ensure transparency, the number of excluded variants is reported, and a sensitivity analysis is presented in the results to illustrate the impact of treating them as misclassifications. Variants from our curated IRD gene list with corresponding data in the Genome Aggregation Database (gnomAD; https://gnomad.broadinstitute.org/) were included for further analysis.

### Extraction and Filtering of ClinVar Variant

To evaluate AlphaMissense performance, clinically classified missense variants were obtained from the ClinVar database, a public archive hosted by the National Center for Biotechnology Information (https://www.ncbi.nlm.nih.gov/clinvar/). ClinVar variants were extracted on February 10, 2025. To account for submitter heterogeneity, review status (star ratings) was noted, and those with assertion criteria provided (1-star rating or higher) were considered. Because reliability varies across submissions and interpretations are continually updated, ClinVar was treated as a useful reference rather than a definitive gold standard. Those missense variants with definitive clinical significance annotations of “Pathogenic,” “Likely pathogenic,” “Benign,” or “Likely benign” were used to compare ClinVar annotations to AlphaMissense scores. Variants labeled as “Uncertain significance,” “Conflicting interpretations of pathogenicity,” or “not provided” or those lacking assertion criteria were excluded from validation analyses. For binary classification, ClinVar annotations were collapsed into 2 categories. Variants labeled as “Pathogenic” or “Likely pathogenic” were grouped as pathogenic, and those labeled as “Benign” or “Likely benign” were grouped as benign.

### Matching of Variants and Concordance Framework

Variants were matched between AlphaMissense and ClinVar using protein-level Human Genome Variation Society nomenclature, based on gene symbol and amino acid substitution. Manual reconciliation addressed discrepancies due to transcript versioning, alternative isoforms, or variant annotation. Concordant variants were those for which both AlphaMissense and ClinVar classifications aligned such that AlphaMissense “likely pathogenic” matched to ClinVar “pathogenic” (true positives), or AlphaMissense “likely benign” matched to ClinVar “benign” (true negatives). Discordant variants were defined as those in which the AlphaMissense classification opposed that of ClinVar. Two main types of discordance were examined: (1) variants predicted to be likely pathogenic by AlphaMissense but classified as benign in ClinVar (false positives) and (2) variants predicted to be likely benign by AlphaMissense but classified as pathogenic in ClinVar (false negatives).

### Blood Sample Collection for Long-Read Sequencing

Molecular genetic testing from Clinical Laboratory Improvement Amendments-certified laboratories for each subject was reviewed. Clinical diagnosis of IRD was based on history, ophthalmology, and audiology findings. One study subject consented for genome sequencing under an approved protocol by the institutional review board at the University of Washington (STUDY00014158). Written informed consent was obtained for a venipuncture to obtain 2 mL of blood for the 1 study subject undergoing long-read sequencing. Genomic DNA was isolated using the MagAttract High Molecular Weight genomic DNA isolation kit (Qiagen). After long-read library preparation, targeted long-read IRD-panel sequencing was carried out with adaptive sampling.^16^

### Sequence Haplotagging, Variant Calling, and Variant Annotation of Long-Read Data

FASTQ files were generated using Dorado and aligned to the generated hard-masked GRCh38 assembly using minimap2.^17^ The binary alignment map file was collated, duplicates marked, and the reads filtered for a minimum alignment quality score of MAPQ 50, and secondary, supplementary, and optical duplicates were removed using SAMtools. Small variants (single nucleotide variants and Indels) were called using PEPPER, and haplotyping was achieved using Margin. Structural variants were analyzed with DeBreak.^18^ The DeepVariant pipeline was used to generate a phased variant call file.^19^ The variant call file files were then annotated with haplotype and phase-block information, variant depth, variant quality, variant effect predictor annotations, ClinVar clinical significance, allele frequency obtained from gnomAD, and CADD score to aid in analysis and prioritization of candidate variants.

### Statistical Analysis

A Welch *t* test analysis was used due to the unequal variance to compare log-transformed allelic frequencies and CADD scores between true positive variants and false positive variants and between true negative variants and false negative variants. These tests evaluated the null hypothesis that no significant differences exist between these variant groups. Bonferroni correction was applied for multiple comparisons, with statistical significance defined as *P* < 1.25 × 10^–2^. Homozygote rates were calculated for each variant group (true positives, false positives, true negatives, and false negatives). The number of variants with ≥1 homozygote reported in gnomAD was divided by the total number of variants in the category for analysis. We evaluated the continuous AlphaMissense score as a classifier by computing area under the receiver-operating characteristic and area under the precision-recall curve using the scikit-learn (v1.5.1) Python package. The comparison was then restricted to only those variants explicitly labeled pathogenic versus variants explicitly labeled benign in the ClinVar database.

### Ethical Consideration

Research protocols were approved by the Institutional Review Board of the University of Washington (STUDY00014158) and were conducted in accordance with the tenets of the Declaration of Helsinki. Informed consent was obtained for a blood draw from 1 study subject who underwent research long-read sequencing.

## Results

### Variant Classification and AlphaMissense Prediction Performance Compared to ClinVar

**Figure 1:**
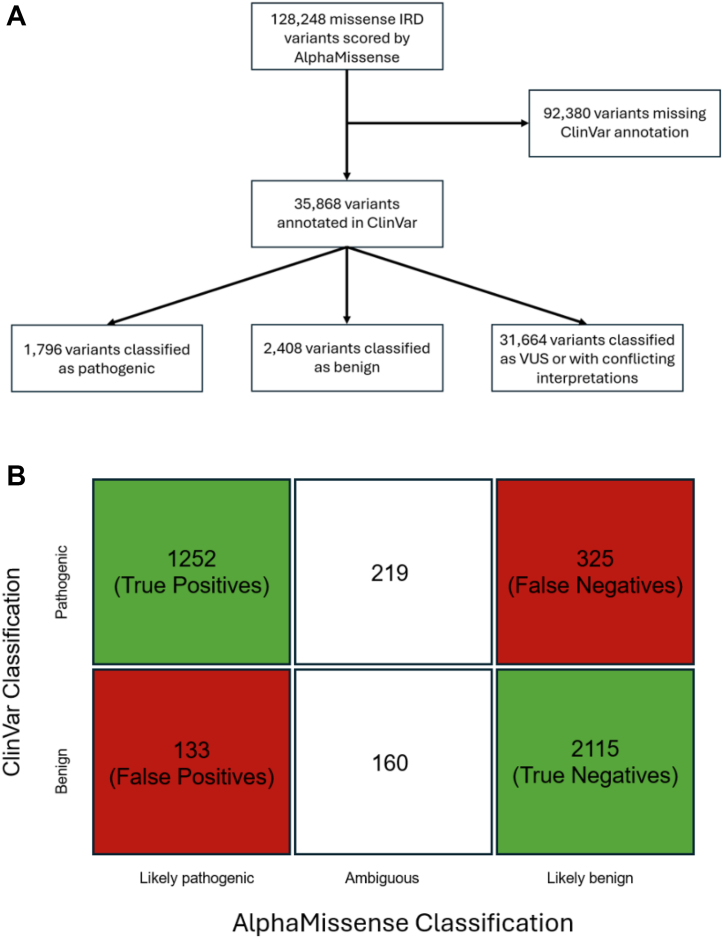
Overview of variant filtering and comparison of AlphaMissense predictions with ClinVar annotations. (**A**) Flowchart outlining the variant filtering strategy used in this study. (**B**) Comparison of variants that were classified as likely benign/benign or likely pathogenic/pathogenic in ClinVar and their corresponding classification by AlphaMissense to calculate parameters of true positive/negative and false positive/negative. IRD = inherited retinal disease; VUS = variants of undetermined significance.

We identified a total of 128 248 missense variants across 107 genes associated with IRDs present in gnomAD. For each variant, we extracted clinically relevant metrics such as allele frequency, homozygote count, and CADD score, as well as precomputed missense variant pathogenicity scores using AlphaMissense. Among these variants, AlphaMissense predicted 12 248 (9.6%) variants to be ambiguous, 89 408 (69.7%) to be benign, and 26 592 (20.7%) to be pathogenic. We then evaluated the predictive performance of AlphaMissense by comparing its pathogenicity classifications against ClinVar annotations. Of the 128 248 missense variants, ClinVar classified 1796 variants as pathogenic, 2408 as benign, and 31 664 as VUS or with conflicting interpretation (Fig 1A). The remaining 92 380 variants were unannotated in ClinVar. We focused our analysis on the 4204 variants with definitive pathogenic or benign classifications.

Among the 1796 ClinVar-pathogenic variants, AlphaMissense correctly predicted 1252 (69.7%) as pathogenic (true positives), 325 (18.1%) as benign (false negatives), and 219 (12.2%) as ambiguous. Of the 2408 ClinVar-benign variants, 2115 (87.8%) were correctly predicted as benign (true negatives), 133 (5.5%) as pathogenic (false positives), and 160 (6.6%) as ambiguous (Fig 1B). Those variants classified as ambiguous were excluded from calculation of sensitivity and specificity. After exclusion, AlphaMissense achieved a sensitivity of 79.4% (95% confidence interval 77.3–91.3), a specificity of 94.1% (95% confidence interval 93.0–95.0), and a false discovery rate (FDR) of 9.6% (95% confidence interval 8.2–11.3) across all the IRD gene variants. The area under the receiver operating characteristic curve for this model was 0.83, and area under the precision–recall curve was 0.87. Of the 4204 ClinVar-classified variants analyzed, 379 (9.0%) were assigned scores within the ambiguous range and were excluded from the primary performance assessment. When these variants were instead treated as errors in a sensitivity analysis, sensitivity decreased from 79.4% to 69.7%, specificity from 94.1% to 87.8%, and the FDR increased from 9.6% to 18.9%. Performance estimates reflect concordance with ClinVar labels rather than biological truth. Per-gene metrics were also calculated for the 107 IRD-associated genes. In examination of the top 20 genes associated with IRDs, the average sensitivity was 83.2%, average specificity was 93.3%, and the FDR was 2.5%. Notably, for 8 of the top 20 genes, including the top 4 implicated IRD genes, AlphaMissense achieved a specificity of 100% (Table 1). We next examined the basis for false positive and negative predictions by evaluating population-based variant metrics.

**Table 1:** Sensitivities and Specificities of the AlphaMissense Predictions for the 20 Most Commonly Implicated IRD Genes

Gene	TP	FN	TN	FP	Sensitivity (95% CI)	Specificity (95% CI)	Sensitivity Notes	Specificity Notes
ABCA4	217	80	22	0	73.1% (95% CI 67.7–77.8)	100.0% (95% CI 85.1–100.0)		No false positives observed; 100.0% (95% CI 85.1–100.0) (TN = 22)
USH2A	97	46	118	0	67.8% (95% CI 59.8–74.9)	100.0% (95% CI 96.8–100.0)		No false positives observed; 100.0% (95% CI 96.8–100.0) (TN = 118)
RPGR	1	1	34	0	50.0% (95% CI 9.5–90.5)	100.0% (95% CI 89.8–100.0)		No false positives observed; 100.0% (95% CI 89.8–100.0) (TN = 34)
PRPH2	29	8	1	0	78.4% (95% CI 62.8–88.6)	100.0% (95% CI 20.7–100.0)		No false positives observed; 100.0% (95% CI 20.7–100.0) (TN = 1)
BEST1	50	10	6	1	83.3% (95% CI 72.0–90.7)	85.7% (95% CI 48.7–97.4)		
RS1	23	1	7	0	95.8% (95% CI 79.8–99.3)	100.0% (95% CI 64.6–100.0)		No false positives observed; 100.0% (95% CI 64.6–100.0) (TN = 7)
RP1	5	1	28	1	83.3% (95% CI 43.6–97.0)	96.6% (95% CI 82.8–99.4)		
RHO	29	4	5	1	87.9% (95% CI 72.7–95.2)	83.3% (95% CI 43.6–97.0)		
CHM	1	0	24	3	100.0% (95% CI 20.7–100.0)	88.9% (95% CI 71.9–96.1)	No false negatives observed; 100.0% (95% CI 20.7–100.0) (TP = 1)	
CRB1	44	26	14	1	62.9% (95% CI 51.1–73.2)	93.3% (95% CI 70.2–98.8)		
PRPF31	1	0	0	0	100.0% (95% CI 20.7–100.0)		No false negatives observed; 100.0% (95% CI 20.7–100.0) (TP = 1)	
MYO7A	83	3	29	5	96.5% (95% CI 90.2–98.8)	85.3% (95% CI 69.9–93.6)		
OPA1	1	0	13	3	100.0% (95% CI 20.7–100.0)	81.2% (95% CI 57.0–93.4)	No false negatives observed; 100.0% (95% CI 20.7–100.0) (TP = 1)	
CNGB3	8	2	18	1	80.0% (95% CI 49.0–94.3)	94.7% (95% CI 75.4–99.1)		
RPE65	59	5	5	1	92.2% (95% CI 83.0–96.6)	83.3% (95% CI 43.6–97.0)		
EYS	9	4	43	1	69.2% (95% CI 42.4–87.3)	97.7% (95% CI 88.2–99.6)		
GUCY2D	19	1	13	0	95.0% (95% CI 76.4–99.1)	100.0% (95% CI 77.2–100.0)		No false positives observed; 100.0% (95% CI 77.2–100.0) (TN = 13)
PROM1	0	3	5	1	0.0% (95% CI 0.0–56.1)	83.3% (95% CI 43.6–97.0)		
CNGA3	50	10	15	0	83.3% (95% CI 72.0–90.7)	100.0% (95% CI 79.6–100.0)		No false positives observed; 100.0% (95% CI 79.6–100.0) (TN = 15)
RDH12	28	6	2	0	82.4% (95% CI 66.5–91.7)	100.0% (95% CI 34.2–100.0)		No false positives observed; 100.0% (95% CI 34.2–100.0) (TN = 2)

CI = confidence interval; FN = false negative; FP = false positive; IRD = inherited retinal disease; TN = true negative; TP = true positive.

### Population-Based Metrics Discriminate Misclassified Variants

Variant characteristics of higher allelic frequencies, lower predicted functional impact scores (such as CADD scores), and the presence of multiple homozygotes present in the population are usually associated with those that impact benign functionality. Pathogenic variants, on the other hand, are rarely observed, have higher functional impact scores, and are generally absent in a homozygous state in the population.^20^, ^21^, ^22^ This prompted us to examine the variant-level characteristics to determine if this could improve the predictive indices of AlphaMissense of the false positive rate of 5.9% and false negative rate of 20.61%.

**Figure 2:**
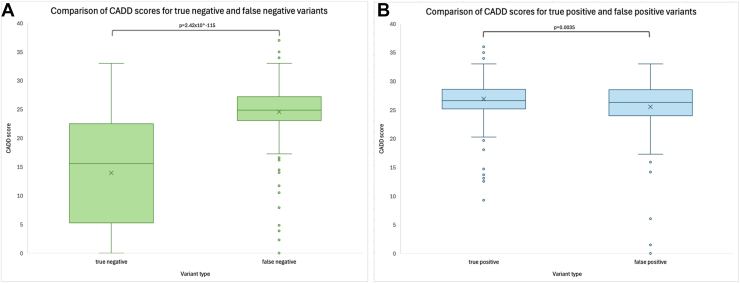
Box and whisker plots comparing the CADD scores can discriminate between variants that were correctly and incorrectly classified by AlphaMissense. (**A**) Combined Annotation Dependent Depletion score was compared between true negatives and false negatives, which were found to be statistically significant. (**B**) Comparison of CADD scores for true-positive and false-positive variants also demonstrated a statistically significant difference. CADD = Combined Annotation Dependent Depletion.

When comparing the allelic frequency metrics between true positive and false positive variants, we found the mean allele frequency for true negatives (3.03 × 10^–2^土1.19 × 10^–1^) was higher than for false negatives (3.35 x 10^–5^土1.41 × 10^–4^), which was statistically significant (*P* = 1.01 × 10^–30^). Conversely, for true positives the mean allele frequency (1.96 × 10^–5^土1.38 × 10^–4^) was lower than for false negatives (3.82 × 10^–3^土2.16 × 10^–2^), although this difference did not receive statistical significance when accounting for multiple comparisons (*P* = 4.46 × 10^–2^). Comparison of the functional impact of genetic variants on protein function using CADD scores demonstrated that false negatives had a higher mean score (24.55土5.661) than true negatives (13.93土9.094), which was statistically significant (*P* = 2.42 × 10^–115^, Fig 2A), whereas true positives had a higher mean score (26.93土2.785) than false positives (25.53土5.35), which also reached statistical significance (*P* = 3.50 × 10^–3^, Fig 2B). Finally, we found the homozygote count was also discriminating with ≥1 homozygote present in gnomAD in 47.75% of true negatives and 30.08% of false positives, whereas the rate was substantially lower in false negatives at 4.31% and in true positives at 1.51% (Table 2). Homozygote rates were 1.51% for true positives, 30.08% for false positives, 47.75% for true negatives, and 4.31% for false negatives (Table 2). These findings show that ancillary metrics of allele frequency, CADD score, and homozygosity in a control population may provide additional interpretative insight when coupled with AlphaMissense predictions. The next section examines the role of this in a major class of discordant classifications in IRD genes: hypomorphic variants.

**Table 2:** Homozygote Counts and Rates for True Positive, False Positive, True Negative, and False Negative Variants

Variant Type	Number of Variants with **≥**1 Homozygote Reported	Total Number of Variants	Homozygote Rate (%)
True positive	19	1252	1.51
False positive	40	133	30.08
True negative	1010	2115	47.75
False negative	14	325	4.31

### Hypomorphic Variants in *ABCA4* Reveal Limitations of AlphaMissense Pathogenicity Predictions

Current variant classification frameworks based on the American College of Medical Genetics and Genomics guidelines^22^ assume high penetrance consistent with monogenic diseases, so the low/incomplete penetrance exhibited by hypomorphic variants creates a challenge in assessing their pathogenicity using these classical guidelines, which we sought to investigate with AlphaMissense. Hypomorphic variants have best been studied in IRDs in the context of *ABCA4*-associated Stargardt disease.^23^, ^24^, ^25^ When evaluating the discordant designations with AlphaMissese in *ABCA4*, we found that of the 9 well-characterized hypomorphic variants (Table 3), AlphaMissense only correctly scored one of them, the well-established Gly1961Glu variant,^26^ as likely pathogenic. Gly1961Glu variant had an allelic frequency of 3.41 × 10^–3^ and 44 homozygotes in gnomAD along with a CADD score of 26.2. The remaining 8 had median allelic frequency of 1.36 × 10^–3^, 5 homozygotes, and CADD score of 25 that were all in the same range as a Gly1961Glu variant that was deemed pathogenic by AlphaMissense. In these cases, although the allelic frequency is higher than would be expected for a pathogenic variant, the lack of homozygotes in many of the variants and the high CADD score would be consistent with a pathogenic classification once other metrics are incorporated with the AlphaMissense prediction.

**Table 3:** Variant Parameters for Well-Characterized Hypomorphic Variants in ABCA4

Missense Variant	AlphaMissense Score and Classification	gnomAD v4 Allelic Frequency	Homozygote Count	CADD Score
Gly863Ala	(0.343) Ambiguous	7.06 × 10^–3^	46	31.0
Ala1038Val	(0.164) Benign	1.85 × 10^–3^	7	19.9
Pro1486Leu	(0.201) Benign	3.79 × 10^–5^	0	24.8
Thr1526Met	(0.366) Ambiguous	1.23 × 10^–4^	0	25.2
Ile1562Thr	(0.180) Benign	1.56 × 10^–3^	3	22.9
Asn1868Ile	(0.223) Benign	5.58 × 10^–2^	2989	23.3
Gly1961Glu	(0.853) Pathogenic	3.41 × 10^–3^	44	26.2
Arg2030Gln	(0.106) Benign	4.77 × 10^–4^	0	27.2
Arg2107His	(0.326) Benign	1.15 × 10^–3^	17	29.3

CADD = Combined Annotation Dependent Depletion; gnomAD = Genome Aggregation Database.

**Figure 3:**
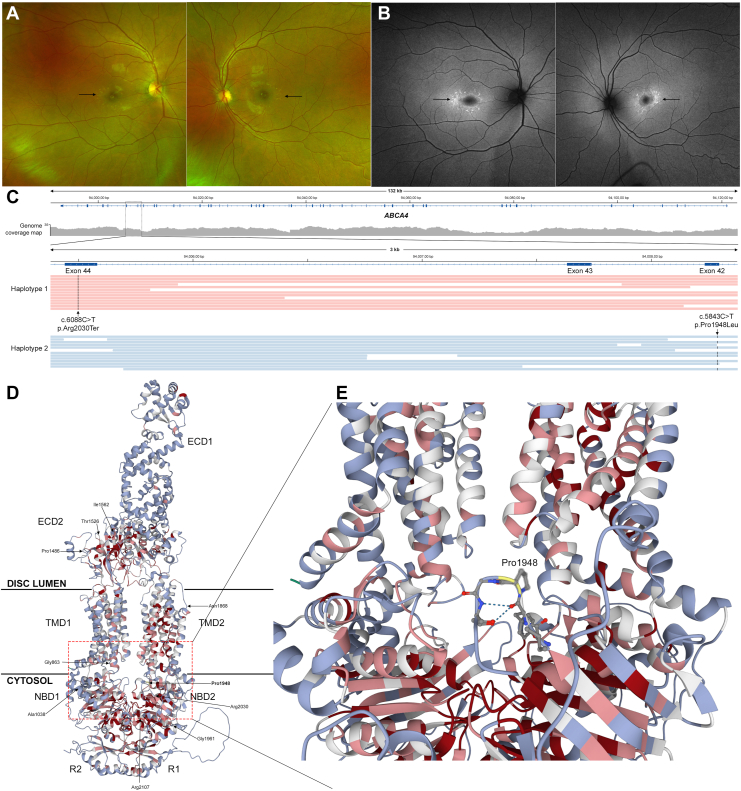
Multimodal and long-read sequencing identifies a potential hypomorphic variant that may be misclassified by AlphaMissense due to its structural feature limitations. (**A**) Color fundus photos reveal macular flecks (black arrows) in both eyes suggestive of a late-onset Stargardt disease phenotype. (**B**) Fundus autofluorescence photos demonstrate hyperautofluorescence of the flecks (black arrows) in both eyes, but relative retinal sparing otherwise. (**C**) Long-read sequencing of this patient provided complete genomic coverage of the *ABCA4* locus and partitioning of the reads into haplotypes for phased variant analysis, which demonstrated that in *trans* to the clinically identified variant, Arg2030Ter, was a potential hypomorphic variant, Pro1948Leu. (**D**) The topological diagram of ABCA4 was used to highlight the location of the known hypomorphic variants as well as the identified Pro1948Leu variant, (**E**) which is in a disordered loop region located in the NBD at the junction of the TMDs. ECD = extracellular domain; NBD = nucleotide-binding domain; R1 and R2 = regulatory domains; TMD = transmembrane domain.

The underperformance of AlphaMissense for hypomorphic variants can affect the prioritization of missense variants as potentially disease-causing, which can preclude a molecular diagnosis. With the ability to produce phased genomic data sets using long-read sequencing^16^ and aid in reclassification of VUS,^27^ the ability to assign potential functional impact can now provide a complete molecular diagnosis without the need for familial segregation. An illustrative example is a 23-year-old female with bull’s eye maculopathy (Fig 3A, B) that was consistent with a milder late-onset macular disease compared with classic forms of *ABCA4*-associated Stargardt disease. Her clinical genetic testing was indeterminate, as it disclosed a single pathogenic nonsense mutation in *ABCA4* (c.6088C>T, p.Arg2030Ter) but no second causative variant. This subject underwent targeted long-read sequencing, and a second candidate variant, c.5843C>T, p.Pro1948Leu, was identified in *trans* to the clinically identified variant (Fig 3C). The potential second variant was predicted to be benign by AlphaMissense, which may be due to the location of this variant as many other hypomorphic variants lie in unstructured regions (Fig 3D, E). Examination of the variant metrics showed that this variant had an allelic frequency of 3.93 × 10^–2^, 1534 homozygotes, and CADD score of 23.5, which is in the range of the other hypomorphic variants in *ABCA4,* such as the frequent hypomorph Asn1868Ile.

## Discussion

Determining the pathogenicity of variants in genes implicated in IRDs remains a diagnostic challenge with clinical implications. Establishing a confirmed molecular diagnosis through accurate classification of variants can provide diagnostic clarity, particularly for VUS. This carries importance in providing accurate familial recurrence risks and may qualify patients for emerging clinical treatment trials. Whereas *in vivo* and *in vitro* experiments are well-standardized methods to confirm variant pathogenicity, they cannot be reliably done in most cases. Deep learning variant predictors, like AlphaMissense, improve functional interpretation by using nonlinear patterns directly from sequence data. AlphaMissense utilizes a transformer-based deep neural network that integrates protein structure and evolutionary context to assess the pathogenicity of amino acid substitutions. The key advantage is its incorporation of structural context and genome-wide predictions, although these predictions have limitations, being based on AlphaFold2. Variants in structured regions are associated with higher pathogenicity scores and can lead AlphaMissense to false positive predictions in structured regions and false negative predictions in disordered regions.^10^ Additionally, AlphaMissense incorporates allelic frequencies into its predictions and is more likely to classify variants with lower frequencies as pathogenic and variants with higher frequencies as benign. In systematically comparing AlphaMissense to ClinVar, which compiles variant-level interpretations of clinical significance submitted by diagnostic laboratories, researchers, and expert panels,^28^ we provide a framework for interpreting these predictions for the most clinically relevant IRD-causing genes.

Our results indicate that AlphaMissense is useful for correctly predicting a variant as pathogenic. For the dataset included in this study, AlphaMissense achieved a specificity of 94.1%, and for 4 of the 5 most commonly implicated genes in IRDs, AlphaMissense demonstrated a specificity of 100% (Table 1). Pathogenic variants in these 5 genes have been implicated in 44% of all IRD cases.^29^ The low false-positive rate of AlphaMissense pathogenicity predictions for these 5 genes indicates it could be extremely useful in correctly predicting pathogenic variants in a large portion of IRD cases. A specific example of this is a patient with Usher syndrome. Genetic testing revealed 2 heterozygous variants in the *USH2A* gene, one of which, c.9921T>G, p.Cys3307Trp, was initially declared a VUS on initial clinical testing, and thus the testing was nondiagnostic. AlphaMissense predicted this variant to be pathogenic with a pathogenicity score of 0.924. The variant was recently reassigned in ClinVar as pathogenic. This example highlights the potential of AlphaMissense to expand understanding of missense variants implicated in IRDs by accurately predicting disease-causing variants as pathogenic, particularly those without definitive annotations in current databases.

The sensitivity of AlphaMissense predictions for the entire dataset was 79.39%, and the sensitivity for the 5 most commonly implicated genes in IRDs was 73.1%, indicating AlphaMissense may not be as advantageous for classifying variants as potentially benign. Due to the high false-negative rate demonstrated in the dataset, it is important to thoroughly evaluate a variant predicted as benign by AlphaMissense. As we detail for *ABCA4*, one of genes with the most well characterized hypomorphic variants, many of which fall in unstructured regions (Fig 3C) where the predictive capability of AlphaMissense is limited. To refine the predictive capabilities of AlphaMissense to better refine such incorrect predictions, allelic frequencies, homozygote counts, and CADD scores were compared between true-negative and false-negative variants and between true-positive and false-positive variants. Our results aligned with the expected trends that benign variants are likely to have higher allelic frequencies, lower CADD scores, and reported homozygotes, whereas pathogenic variants tend to have lower allelic frequencies, higher CADD scores, and no reported homozygotes.^20^, ^21^, ^22^ An exception is false positive variants that demonstrate high average CADD scores, though a low CADD score in a predicted pathogenic variant could raise suspicion for a false positive. Based on our findings, a false negative should be suspected if a predicted benign variant has a lower allelic frequency, higher CADD score, and does not have a reported homozygote. Conversely, a false positive should be suspected by a higher allelic frequency, lower CADD score, or presence of homozygotes. While exceptions such as true positives and true negatives that demonstrate these patterns exist, this framework is useful in raising suspicions for variants that require further analysis for their validation of their pathogenicity status.

Of note from this study, 92 380 variants scored by AlphaMissense were not classified in ClinVar. When examining these, we found that 1755 of the 31 664 variants classified as conflicting/VUS and 19 452 of the 92 380 variants without a ClinVar annotation were predicted to be pathogenic by AlphaMissense (Fig 1B). Given the FDR of 9.6%, it is possible that upward of 90% of these variants without definitive annotations in ClinVar are potentially pathogenic. This finding highlights the potential of AlphaMissense to uncover novel missense variants implicated in IRD pathogenesis that are currently unclassified.

An important consideration when interpreting our results is the reliance on ClinVar as the reference standard for benchmarking AlphaMissense. ClinVar is a widely used resource, but it is not a definitive gold standard. Its classifications are derived from multiple submitters with heterogeneous levels of evidence, ranging from single-laboratory reports to expert panel reviews. This heterogeneity is reflected in the review status (star rating) system, which provides an indication of confidence but does not eliminate variability or potential errors. In addition, ClinVar is a dynamic database, with variant interpretations updated over time as new evidence emerges. As such, our analysis represents a snapshot of concordance with ClinVar annotations at the time of data extraction rather than immutable biological truth. Performance estimates should therefore be interpreted as reflecting agreement with ClinVar labels, rather than absolute measures of predictive accuracy. This context emphasizes the importance of interpreting benchmarking studies alongside functional assays, segregation analyses, and clinical correlation when evaluating the pathogenicity of missense variants. Another consideration is that our benchmark set was restricted to variants present in gnomAD. This choice provided consistent allele frequency and homozygote data for all variants, which was important for downstream analyses. This restriction necessarily excluded some ultrarare pathogenic variants not captured in gnomAD, but the overall performance estimates should be interpreted considering this sampling frame.

This work highlights AlphaMissense as a new approach to guide our designation of newly identified genetic variants as potentially disease-causing or not for IRDs. This will be of increased importance as we are able to carry out haplotype-specific analysis of variants in the proband alone with long-read sequencing to assign pathogenicity and provide complete molecular diagnostics.^27^ In this work, we highlight that AlphaMissense has a specificity of 94.1% but a sensitivity of 79.4%. This should help guide clinicians and researchers working in IRDs to be aware of the higher false negative predictions. As we outline, this may be due to the complexity of variants such as hypomorphic variants, as they often produce subtle protein level changes and have other metrics such as high allelic frequency that can cloud their predictive metrics. However, incorporating allelic frequencies, homozygote data, and CADD scores into consideration can help refine AlphaMissense predictions and provide a method to better classify the pathogenicity of missense variants.

## Data Availability Statement

The AlphaMissense and additional data tools (i.e. CADD) used in this work are publicly accessible. Genome sequencing data are not publicly available due to privacy and patient anonymity issues. Access to the genome sequencing data will require an IRB-approved collaboration and Data Usage Agreement.
